# ‘None of Them Know Me’: A Qualitative Study of the Implications of Locum Doctor Working for Patient Experience

**DOI:** 10.1111/hex.14156

**Published:** 2024-08-01

**Authors:** Jane Ferguson, Gemma Stringer, Kieran Walshe, Ailsa Donnelly, Christos Grigoroglou, Thomas Allen, Evangelos Kontopantelis, Darren M. Ashcroft

**Affiliations:** ^1^ Health Services Management Centre The University of Birmingham Birmingham UK; ^2^ Alliance Manchester Business School The University of Manchester Manchester UK; ^3^ NIHR School for Primary Care Research, Centre for Primary Care and Health Services Research University of Manchester Manchester UK; ^4^ Manchester Centre for Health Economics, Division of Population Health, Health Services Research and Primary Care The University of Manchester Manchester UK; ^5^ Danish Centre for Health Economics University of Southern Denmark Odense Denmark; ^6^ NIHR School for Primary Care Research, Centre for Primary Care, Division of Population Health, Health Services Research and Primary Care The University of Manchester Manchester UK; ^7^ Division of Informatics, Imaging and Data Science University of Manchester Manchester UK; ^8^ NIHR Greater Manchester Patient Safety Research Collaboration, Division of Pharmacy and Optometry, Faculty of Biology Medicine and Health University of Manchester Manchester UK

**Keywords:** continuity of care, locum doctors, patient experience, patient safety, PPI, qualitative

## Abstract

**Introduction:**

There have been some concerns about the impact of temporary doctors, otherwise known as locums, on patient safety and the quality of care. Despite these concerns, research has paid little attention to the implications of locum working on patient experience.

**Methods:**

A qualitative semi‐structured interview study was conducted with 130 participants including locums, people working with locums and patients with experience of being seen or treated by locums. Analysis was conducted using a reflexive thematic approach and abductive analysis to position themes against wider knowledge.

**Results:**

Three main themes were constructed through analysis: (1) Awareness and disclosure; patients were not always aware if their doctor was a locum, and there was some debate about whether patients had a right to know, particularly if locum working presented quality and safety risks. (2) Continuity and accessibility of care; access was regarded as priority for acute conditions, but for long‐term or serious conditions, patients preferred to see a permanent doctor who knew their history, although it was acknowledged that locums could provide fresh perspectives. (3) Communication and practice; locums and patients described how consultations were approached differently when doctors worked as locums. Patients evaluated their interactions based on how safe they felt with practitioners.

**Conclusion:**

Patients reported that they were unlikely to have continuity of care with any doctors delivering care, regardless of their contractual status. Locums sometimes provided new perspectives on care which could be beneficial for patient outcomes, but for patients with long‐term, complex or serious conditions continuity of care was important, and these patients may avoid or delay seeking care when locums are the only available option.

**Patient or Public Contribution:**

Patients and carers were involved in our study from inception to dissemination. Our Patient and Public Involvement (PPI) forum was involved throughout project design and planning and gave us feedback and guidance on research materials and outputs (e.g., study protocol, participant information sheets, survey tools, interview schedules, emerging findings). Our PPI forum co‐produced our patient interview schedule, two members of our PPI forum led the patient focus groups and all were involved in analysis of patient interviews. Our PPI Chair was involved in the preparation of this manuscript.

## 1 Introduction

‘Locum’ doctors are doctors who work in temporary positions covering gaps in healthcare rotas and can work in healthcare organisations for periods as short as 1 day or long term for months or years. Internationally, the use of locums has increased [1, 2, 3, 4], partly as a response to global doctor shortages [5] and in the United Kingdom, poor working conditions [6]. In the United Kingdom, all doctors, apart from those in their first year of post qualification training, can work as a locum. Their conditions of employment, responsibilities and scope of practice can differ from the doctors they are replacing, which may influence patient experience [7]. Locums may work in familiar or unfamiliar organisations, and as a consequence of these differences in employment arrangements, continuity of care can vary widely [7].

There have been widespread and sustained concerns among policymakers, healthcare providers and professional regulators about the quality and safety, cost and effective use of temporary doctors [3, 8, 9, 10]. The experiences and perceptions of locum working from the perspectives of locums and those who work with them have been explored elsewhere [11], but the patient voice has been largely absent from the literature. Despite temporary working being a common feature of healthcare, to the authors' knowledge, there are no empirical studies directly examining the implications of locum working for patient experience from the perspective of patients, locums and other healthcare professionals.

Changes in doctors' working patterns have been attributed to unmanageable workloads and burnout, reported by doctors in all areas of the profession [12]. A decline in full‐time working in favour of part‐time, portfolio and locum working [13] combined with persistent understaffing [14] and unprecedented demand for appointments [15] has resulted in detrimental effects for patient access to healthcare and continuity of the doctor−patient relationships [16]. Public satisfaction with access to care is at an all‐time low [17, 18]. Locum doctors play an important part in facilitating access to care when there are staff shortages or gaps in rotas. Greater access and continuity of care are core features of high‐quality healthcare systems and are associated with lower mortality, better health outcomes, higher patient satisfaction and lower cost [13, 19, 20, 21, 22, 23]. However, research examining continuity of care generally excludes locum doctors [24, 25, 26], despite locums making up a significant proportion of the healthcare team [3].

The benefits of continuity of care have been widely demonstrated [20], and timely access to services is also an essential component of patient safety [23]. Patient‐centred care should be aligned with what patients consider important and patients vary in their preference for continuity [27]. While access to and continuity of care can positively affect patient outcomes, they are just two aspects of a well‐functioning healthcare system, and quality of care is a concept that is at its most meaningful when applied to individual users of healthcare [23]. Mortality rates and clinical indicators give only a partial view of the value of healthcare [28] and what matters most to patients can vary depending on individual circumstances [29]. How locums fit into the debate about how to promote access and continuity of care in modern healthcare has not been fully explored in the literature, and the views of patients have not previously been researched.

Recent evidence suggests patients predominantly conceptualise patient safety in the context of what makes them ‘feel safe’, and feelings of safety were intrinsically linked to having trust and confidence in healthcare providers [30, 31]. Trust has been described as a key mechanism by which doctors manage complexity and uncertainty and negotiate their professional roles and identities [32]. There is evidence to suggest that communication and trust make a significant contribution to the health‐seeking behaviours of patients [33]. In general practice, patients' trust in GPs reduced requests for low‐value interventions (such as scans), and trust and continuity between doctors and patients were important for patients to accept the doctors' decisions [7, 34]. However, while there is some evidence to suggest that locums felt they were regarded as less trustworthy by patients and colleagues [11], how patients perceive trust when doctors are temporary is not well understood. Understanding the implications for patients when care is provided by locums is important as patient perceptions about safety can differ from those of healthcare professionals [30]. This research was part of a wider research project that aimed to provide evidence on how locum working arrangements impact quality and safety in primary and secondary care in the English NHS [7].

## 2 Aims

Literature about patient and doctor perspectives and experiences of locum doctor working is sparse. Such data have the potential to provide important information about how patients access care, their experiences of continuity of care, as well as providing insight into the how patients respond when care is provided by locums. The aim of this research was to address this gap by exploring the implications of locum working for patient experience from the perspective of patients, locums and other healthcare professionals.

## 3 Methods

An in‐depth qualitative study design was adopted using semi‐structured interviews and focus groups. Participants were offered the option of participating in a one‐to‐one interview or a focus group. Semi‐structured interview and focus group schedules allowed participants to share their experiences while also providing insights on questions identified by the research team and PPI forum. In this paper, we foreground our findings from exploratory focus groups and one‐to‐one interviews with patients. However, we have also drawn on our entire data set to understand the implications of locum working for patient experience by including perceptions from multiple sources to provide a holistic view. The study received ethical approval from the Health Research Authority (IRAS project ID: 278888; REC reference 20:/NW/0386). All participants provided written informed consent. The Consolidated Criteria for Reporting Qualitative Research (COREQ) guidelines were applied [35] (see Supporting Information).

### 3.1 Recruitment

A qualitative semi‐structured interview study was conducted with 130 participants including locums, people working with locums and patients with experience of being treated by locums (see Table 1 for more details). Locums and people who worked with locums were purposively sampled through 11 organisations, including NHS trusts, primary care practices, statutory NHS bodies and locum agencies. Participants included locums, permanently employed doctors, health professionals (nurses, pharmacists), leads for medical staffing and clinical governance and practice managers. We recruited 42 patients through ‘Research for the Future’, a National Institute for Health and Care Research Clinical Research Network initiative to help recruit participants in health and care research using a ‘consent for approach’ model.

**Table 1:** Characteristics of study participants.

Characteristics	Professionals (*n* = 88)	Patients (*n* = 42)
Mean age^a^ (SD)	46 (11.0)^b^	59 (14.4)^a^
Gender (women)	49 (56)	24 (57)
Ethnicity		
White English	45 (51)^c^	30 (71)^a^
Any other White background	12 (14)^c^	4 (10)^a^
Mixed/multiple ethnic groups	3 (3)^c^	1 (2)^a^
Asian/Asian British	15 (17)^c^	2 (5)^a^
Any other Asian background	2 (2)^c^	0^a^
Black/African/Caribbean/Black British	2 (2)^c^	1 (2)^a^
Other ethnic group	1 (1)^c^	0^a^

*Note:* Data are presented as frequency (%) unless stated otherwise.

^a^ *n* = 38.

^b^ *n* = 81.

^c^ *n* = 80.

### 3.2 Data Collection

Three semi‐structured interviews and focus group guides were developed for use with patients, locums and health professionals working with locums. These schedules were informed by our previous review of the literature relating to quality and safety [10] and our earlier qualitative study [11]. Three experienced qualitative health services researchers (J.F., G.S. and K.W.) conducted 88 interviews with participants who worked in healthcare. With support from two members of the research team, two members of the PPI forum (M.M. and M.S.) carried out five focus groups with 30 patients, and G.S. and J.F. carried out 12 one‐to‐one interviews. Focus groups involved between four and eight participants per group. J.F. and G.S. also carried out 12 one‐to‐one interviews. Qualitative data were collected between March 2021 and April 2022. Focus groups typically lasted an hour, while interviews varied in length from 23 to 171 min. Audio recordings were transcribed verbatim by a professional transcription company and organised into codes and themes using the software package NVivo [36].

### 3.3 Analysis

Analysis was ongoing throughout the data collection period and followed a reflexive thematic approach [37]. The research team and the PPI forum worked together reflexively discussing their biases and their potential impact on the research. Analysis was primarily carried out by J.F., G.S. and K.W., who are experienced qualitative researchers and involved familiarisation with the data by reading all the transcripts; coding the data set and organising all relevant data extracts and generating initial themes to identify broad patterns of meaning across the data set. Key themes and quotes were circulated to the wider research team, which comprised health services researchers, and the PPI forum for comment and discussion. These involved questioning whether the themes answered the research questions and told an authentic story of the data. On the basis of these discussions, themes were either combined, separated or discarded or named or renamed. Finally, the analytical write‐up positioned the themes in relation to existing literature and knowledge using an abductive approach [38], which allowed for the integration of inductive data‐driven coding with deductive interpretation [39].

## 4 Results

Our findings are presented under three themes that examine implications of locum working for patient experience from the perspective of patients and staff. The themes are as follows:

- Awareness and disclosure—views on whether patients should know if their doctor was a locum.
- Continuity and accessibility of care—implications of locum working on continuity and access.
- Implications for practice and patient outcomes—how practice differs when doctors work as locums and the implications for patient outcomes.

### 4.1 Awareness and Disclosure

Patients were not always aware, in both primary and secondary care, whether their doctor was a locum and spoke mainly about their experiences of seeing a locum at their GP practice, where they were more likely to be familiar with permanent doctors. Patients felt it could be difficult to distinguish who was permanently employed or a locum, especially when there were lots of doctors with different working patterns. Patients described interactions with doctors as often being transactional, one‐off consultations with unfamiliar doctors who they perhaps might not see again, despite them being permanently employed at an organisation.

When you see somebody, do you even know if you're seeing a locum? … if you ring up the doctor because you're ill and they say, well, I'll give you an appointment with Dr Whatsit. And you haven't a clue who Dr Whatsit is and you've never seen Dr Whatsit before, Dr Whatsit apparently has never seen your notes either, it's quite muddling.

(Focus Group A, R2)

There were differing opinions among patients and healthcare professionals about whether patients should be informed whether the doctor was a locum. Some patients felt it was important, particularly for those with long‐term, complex or mental health conditions to be given information about who they were seeing, especially if they have not seen that doctor before.

That's very important. Like as a patient, it should be explained under the charter of the service, what the whole purpose is of your treatment, wherever you'll be seen by a junior doctor. So wherever you'll be seen by a locum, or wherever you'll be seen by another specialist.

(Interview 95, Patient)

I think when it's long‐term patients, as it mostly is with mental health, I think they do need to know that it's a locum, because it will be a long‐term relationship usually.

(Interview 86, Clinical Lead Secondary Care)

Patients, locums and healthcare professionals perceived there may be a reluctance to disclose to patients whether their doctor was a locum because of the recognised negative perceptions and prejudice around locum working. Participants described the potential implications of disclosing whether a doctor was a locum for consultations and doctor−patient relationships, health‐seeking/avoidant behaviours and potential adherence to treatment.

Why would you badge it with locum because I think if the reality is if you do use the word locum, I think most patients are familiar with the word locum and they'll see it in a negative way as this is somebody that's not going to know me, this is somebody that's not going to be as close to my history and it'll put those barriers up almost immediately.

(Interview 27, Medical Director)

Sometimes patients don't react in the best way to knowing that it's a locum doctor. Oh no, I want to see a ‘real’ doctor.

(Interview 25, Medical Staffing, Secondary Care)

While some patients felt that patients should know when a doctor was working as a locum, the general consensus amongst healthcare professionals was that patients did not need to know. Healthcare professionals perceived that patients in secondary care were less aware and concerned about whether the doctor was a locum and that they did not need to know because the standards of safety, patient care and governance processes should be the same regardless of the doctor's contract. Some healthcare professionals perceived that patients were not concerned if they were seen by a locum, as long as care was delivered by a doctor who was qualified and capable.

No, I don't think they should or be told. I think the system ought to be robust and safe enough that it doesn't matter.

(Interview 30, Responsible Officer, Secondary Care)

I'm not sure patients do need to know 'cause there's no difference in terms of they've got the required skillset, they've got the required training, they've got the required knowledge, I'm not sure there is a need to know.

(Interview 25, Medical Staffing, Secondary Care)

### 4.2 Continuity and Accessibility of Care

Traditional notions of relational continuity of care or the on‐going therapeutic relationship between a patient and a doctor [40] were often regarded as a thing of the past. Traditional ideas of a ‘family GP’ were recognised as increasingly difficult to sustain in modern healthcare. This was perceived to be a consequence of how general practice was now structured and how GPs approached their working lives, with more GPs choosing to work flexibility.

I desperately want it to all be about seeing the GP and having continuity, and them knowing me and my family inside out. But I do think we're at the stage where that's kind of like a media construct of a GP, it's like a country practice version of a GP. And it hasn't existed for like five decades.

(Focus Group B, R2)

Patients described their experiences of making appointments and typically not being offered a choice about which doctor they saw. Access to a doctor and seeing someone rather than no one meant that patients felt they did not have the option of choosing who they saw. For some patients, access to care was more important than continuity.

You can never book an appointment with your doctor at our surgery. It's as simple as that. So you've got a choice, you have a locum or you don't see a doctor.

(Focus Group B, R2)

Patients generally spoke about continuity of care in two ways: their relationship with the doctors and how well they knew them (relational continuity) and how information about their care was recorded, shared and communicated (management and information continuity). Patients recognised that they were unlikely to been seen by the same doctor at a practice, regardless of whether the practice employed locums, and relational and information and management continuity was regarded as difficult in all practices.

I don't even think our general GPs are familiar anymore … These days I don't even see a regular GP. I end up with them on the end of a telephone … I've never seen the same person and yes, well, a few of them were locums. None of them know me.

(Focus Group C, R3)

Have they read the notes? Do they know me? What continuity am I going to have with this person? How long are they there? Are they there for a week, a month, six months, whatever? Is it worth trying to get a relationship with them?

(Focus Group C, R4)

Patients and healthcare professionals spoke about how continuity of care was generally no longer held by an individual doctor but in their patient records which were maintained electronically by multiple doctors and sometimes across organisations. Given that relational continuity was no longer the norm, good quality management continuity was seen as essential. However, there were concerns that there were inconsistencies and omissions in terms of recording and sharing information and that the technological infrastructure was not robust enough to ensure continuity of information. Furthermore, it was perceived that the temporary nature of locum working meant that locums were not as accountable to their patients or organisations.

One thing that occurred to me is that the whole business of continuity of care seems to be focused on continuity of doctors’ notes … I think one thing I would say is that if I want continuity of care, I want continuity of information passing.

(Focus Group D, R4)

I do have a concern though and maybe even a fear that you don't have the continuity. When you have a rare condition, the one thing you want is continuity or faith in somebody understands the condition that you have, in my case. And that continuity hasn't been there. And it worries me when I see a locum, they only … they can only look at the records and sometimes they're not as up‐to‐date as the last time you went to see the consultant … And it's a fear really and a concern that there isn't … because you'll never see the same locum again. And they're not accountable, they come and they go.

(Focus Group A, R5)

Locum unfamiliarity could increase the likelihood of information being incorrectly recorded or shared. Locums reported that errors could sometimes ‘slip through the net’ or care could be delayed because of discontinuity and unfamiliarity with patients, systems and processes. Implications for patients included delayed treatment or discharge or referral to the wrong service. However, there was a perception that patients were unlikely to come into direct harm because of locum working due to the rest of the team compensating where there were deficits in local knowledge of local procedures, for example.

The drawback is unless you read somebody's notes thoroughly, you won't know them as well, and for some people, that's really important, you've got a very complicated history, you will not know as much as their regular doctor about them and you'll have to be making compromises in terms of the time you spend to educate yourself on them, and the flipside is you may not be familiar with referral pathways, there's a higher risk of you sending them to the wrong place, filling in the wrong form, creating an admin burden for somebody down the line because you didn't get it right.

(Interview 35, Locum GP)

Patients often saw continuity of care as a safety issue and valued continuity when seeking care for complex, serious or long‐term problems and more amenable to seeing a locum for minor acute illnesses that were unlikely to require follow‐up. Patients expressed frustration when they had to recount their detailed medical history to a locum doctor. Locums were regarded as particularly disadvantaged if they had no previous experience of a patient and their medical history and would need to read the notes before or during the consultation, which was frustrating for patients and gave the impression they were not being listened to.

I prefer to see a doctor who knows me because my medical history is complicated. And I think you haven't got time to look at everything … you're not seen as a whole. And that's upsetting … that's my experience is that if they say a locum, I just think they're not going to get the full picture, it's easy to dismiss. Whereas when you see a regular doctor who knows your history, I feel at least I'm being listened to more.

(Focus Group B, R1)

I've got no problem with being seen by a locum for a one‐off first event. …, I think that they should just be dealing with first visits or minor complaints. I don't think they should be standing in for the GPs with people with serious health conditions.

(Focus Group C, R2)

There was concern that a lack of relational, management and information continuity could have a negative impact on patient outcomes for patients with serious or chronic conditions and a perception that the absence of continuity placed additional responsibility on the patient to be an accurate reporter of their history.

I think for some patients, especially patients with a background of dementia, or patients who are acutely delirious, I think it can be quite confusing when you get a different doctor day upon day, and even from the simple tasks like doing a cognitive score, one of the questions is, do you know what my job is? And a patient is more likely to give an accurate answer if they've seen you for the last two days already, as compared to if it's the first time you've walked in the room.

(Interview 23, Locum Secondary Care)

So it concerns me that you've got to spend time going over stuff that, you know, you expect an experienced doctor or a regular doctor that you see, if they're not available, then you've got to go over stuff. And sometimes you forget, oh, I should have mentioned this, should have mentioned that, which is relevant.

(Focus Group A, R5)

Healthcare staff, patients and carers for people with long‐term or mental health conditions described the importance of continuity of care and the implications for patients when knowing relationships were absent. Not knowing seemingly minor details about a patient could have significant ramifications for their mental health and for those caring for them. For psychiatric inpatients, a lack of patient−doctor relationship could result in locum psychiatrists being more risk averse and patients being detained for longer than necessary. Being treated by a succession of short‐term locums was regarded as being detrimental for this group of patients.

Sometimes where locums go wrong, my mum has mental health issues where she gets paranoid … My regular GP knows that if he decides to change the brand or the medication … he always lets me know beforehand … The locums didn't do that and they suddenly sent a different brand and different colour and shape. I had three weeks stress with my mum because she was convinced that this is not the right med …. she was going through all these conspiracy theories about this is the cheaper options to save money or they want to kill old people off.

(Focus Group A, R6)

If they're just coming in for a three‐month period, they aren't going to take a risk on somebody in discharging them in the last week of their contract. They're not going to do that. To change a patient's pathway, there's no positives, I don't think in mental health services for patients with locums. I can't see it. I can't see any.

(Interview 86, Clinical Lead, Secondary Care)

Being able to trust their doctor was a significant issue for some patients, and locums were considered an ‘unknown quantity’. Patients who avoided making appointments with doctors they did not know or trust were concerned that locums would not know their history and this could have negative implications for their care. Patients with long‐term and/or mental health conditions reported delaying seeking nonurgent care when their regular GP was not available.

I refused to see locums anymore after that experience [over prescription of antibiotics] … It shouldn't happen. I'll readily wait until I can see somebody I know and can trust.

(Focus Group D, R3)

Unless it was a real, real emergency, I'd wait till my regular GP was in … I just had to wait'til he'd come back off sabbatical, they said he won't be back for four to six weeks … until I knew my doctors was back, I wouldn't make an appointment.

(Interview 74, Patient)

Whenever I try and make an appointment, they do say that you can be seen by a different GP, but I insist I'd rather be seen by my regular GP, who's aware of my treatment, who's aware of my plans. So I insist and I request that, otherwise I don't want to be making an appointment … I don't feel comfortable, or there's trust issues … 

(Interview 95, Patient)

A patient questioned the cost effectiveness and efficiency of seeing a locum and having to explain their history compared to a shorter appointment with a familiar doctor.

If I go in and take up 40 min of a locum's time trying to get my point across, telling him the answers to all these things he's asking, how many patients could he have seen? Four patients. If I go and see my long‐term regular doctor, we could probably get it done in 10 to 15 maybe. I don't know. I think there's definitely … there's an investigation or something needs to be looked at with regard to time management, patients seen and cost effectiveness.

(Interview 76, Patient)

### 4.3 Implications for Practice and Patient Outcomes

Locums described how their practice sometimes differed in comparison to permanent doctors, and patients and other healthcare professionals corroborated this. Locum working was described as influencing how doctors practised in a number of ways, which had consequences for patient pathways and outcomes. Locums could be more autonomous and less constrained by organisational policies and procedures, but they also recognised a need to compensate for a lack of doctor−patient relationships and described how they approached consultations differently from permanent doctors. Locums were aware that they lacked the advantage of previous encounters and they could be perceived negatively; as a result, locums described having to work harder to ‘win trust’ to make patients feel safe and heard.

It's like being an actress sometimes doing our job, you know, you have to put it all on in some ways … I think when I've been a long‐term doctor, a long‐term salaried GP, you can see that they trust you from the outset because you've helped them before I think and it all went well. I think so. So yeah, you probably have to try that bit harder as a locum, you know, you have to give them some eye contact and listen to them while you're looking in their eyes, that kind of thing, to build up some kind of idea that you are genuinely interested in their problem.

(Interview 90, Locum GP)

Locums were also aware that they were more likely to be complained about by patients, which could have implications for their employment. These factors influenced consultations and communication and potentially patient care and outcomes with some locums reporting being more likely to practise defensively and offer tests and investigations to avoid patient complaints.

Being risk‐averse and practising defensive medicine usually means more tests, more referrals, whereas holding risk tends to be disadvantageous for you as a locum because what's the benefit to you of not doing that. You're benefiting the system by rationing resource, the patient won't thank you, and again as a locum, you don't need to have the patients coming out singing your praises, but it certainly helps.

(Interview 35, Locum GP)

Perhaps as a consequence or the change in relationship dynamic between the locum, the patient and the organisation, locums and patient participants perceived locum practice as being less constrained and influenced by the norms, politics and policies of the organisation in comparison to permanent doctors. This reduction in organisational accountability and increased autonomy had a number of benefits for patients, including locum GPs being able to spend more time with patients and/or order medications and referrals that other doctors in the organisation may not have ordered because of financial constraints for example.

That doctor, through that line of questioning and not having any sort of prior history … may have had a chance to look at my notes beforehand, ordered the right tests and didn't feel constrained in that practice about what tests that they could order. And someone subsequently … because when you get referred to hospital, the consultant said that that doctor was very much on the ball. And, of course, that's a change to lifelong medication. And literally within a month of the medication kicking in, it transformed my life.

(Focus Group A, R1)

Another locum described how a lack of responsibility and accountability for the consequences of their practice meant that it was easy to become disengaged. Doctor disengagement from patient was regarded as a disadvantage to patients whilst also damaging the reputation of locums.

I think it is very easy, as a locum, to be very lack‐lustre, to not engage … where the consequences of your actions, as long as they're not huge, horrific complaints, quite often don't come back to you. I think it's very easy to be that sort of individual, and that's where you get these reputations of locums being a liability.

(Interview 23, Locum Secondary Care)

Patients spoke about how locums had provided fresh perspectives on their condition that their regular GP may not have considered. Locums not having preconceived ideas about a patient or knowing their history may be an advantage for some patients as the locum may not be building on a previous treatment plan but instead considering something new. In this sense, ‘disruptive discontinuity’ was a positive. However, not having a follow‐up with the same doctor meant there was a missed opportunity for the locum and the patient to assess outcomes.

In my case, continuity was damaging because there's kind of an assumption that if you know the person and you know the personality, then when they come in with certain issues, you dismiss it because, well, it's just them isn't it, I know what they're like, they'll exaggerate slightly, they'll do this. And actually sometimes seeing a locum or somebody who doesn't know you makes them just look at the medical stuff, just look at the factual stuff. And in my instance, that was life‐changing. You know, that was literally life‐changing because I got somebody who didn't dismiss it, who didn't sort of go, well, it's just him and his problem he comes to the GP practice regularly, you know. So I think we can invest too much in this kind of … version of, you know, see the same GP for all your life and everything's magically okay. It really isn't.

(Focus Group B, R2)

I have benefited from some locum doctors in the past because they've brought a fresh pair of eyes and perspective on it … But then there's the follow‐up session again because then you're back to that, you're not going to see them again. It's mainly the inconsistency that gives you the uncertainty.

(Focus Group E, R1)

## 5 Discussion

The temporary nature of locum working had implications for patient outcomes, doctors' practice, doctor−patient relationships and how patients received and accessed care. Locums, while enabling access to services, were potentially detrimental for continuity of care and the health‐seeking behaviours of patients to whom continuity mattered. Locums were, either as a result of defensive practice or being less constrained by organisational protocols, able to offer alternative perspectives and pathways which sometimes led to significant improvements in health and patient satisfaction.

Our research found that patients experienced many of their interactions with doctors as one‐off transactional encounters, regardless of whether their doctor was a locum. Pressures on the NHS have been described as eroding the doctor−patient relationship: essential for good medical care [41]. It has long been understood that changes in how care is organised can erode trust [42]. Modern working practices, in response to poor working conditions and subsequent doctor shortages, increasingly place the interests of patients in direct conflict with how care is managed and arranged [43]. The situation is made more difficult by restrictions on patient choice. We found, as others have [44], that patients were offered a choice between rapid access to care and seeing their regular doctor or not offered a choice at all about who they saw.

Whether or not the doctor was a locum was sometimes not communicated to patients and justified because the assumption was that standards of care were the same, despite evidence to suggest systems of governance and standards of safety were not as robust for locums [10, 11, 45, 46]. Participants in our study argued that withholding doctor employment status was warranted because of potential negative responses and the consequences of these for ongoing care. However, ongoing therapeutic relationships are important to some patients [25, 44, 47, 48], and evidence suggests that withholding information from patients disempowers them and erodes trust [49]. Given that patients were sometimes less trusting of locums, it seems paradoxical then that healthcare staff withheld information to build trust.

While patient experiences of locum doctor interactions were sometimes indistinguishable from their experiences with permanently employed doctors, who they were also unlikely to know, differences were evident for patients who valued relational continuity. Locums, and especially short‐term locums, were less able to acquire the accumulated knowledge about patients and the local health economy that facilitated continuity of care and enabled its associated benefits [50]. Patients looked to their doctors as the ‘keepers of their story’ and felt that repeating their history could be frustrating, risked omissions and relied on patient memory and health literacy [51]. It could be argued that if relational continuity is less likely, good management continuity is essential. We know that when information transfer and technology for accessing health records is insufficient, patient outcomes are negatively affected, and patient confidence in professionals is reduced [52, 53]. It is often assumed that in the absence of relational continuity, doctors are interchangeable and any competent doctor can deliver continuous care through good management of information and clinical notes [54, 55]. However, recent evidence suggests that this is not the case and without knowledge and experience of local health systems and even experienced and knowledgeable doctors can fail to provide good care for their patients [55].

Disruptive discontinuity was cited as an opportunity for highlighting areas of improvement and was sometimes positive for patient care, which reflects positive patient outcomes on second opinions in other research [56]. However, dividing care to facilitate continuity by channelling patients with complex or long‐term conditions to permanent doctors and acute patients to locums may further erode the continuity of care as well as impact the professional development of locums. What is perhaps more likely to improve care is better organisational support for doctors who are working in unfamiliar environments.

Locums are fully qualified doctors from heterogeneous backgrounds who sometimes also hold a permanent position. Yet, locums were sometimes perceived by patients and other healthcare professions to be less experienced, less capable and less committed than permanent doctors. Negative perceptions of locums and lack of trusting relationships meant some patients in our study avoided consultations with locums, delayed accessing care or made a second appointment with their regular doctor after being seen by a locum. Trust contributes to improvements in health outcomes [57], and lack of trust can result in negative perceptions of continuity of care, poorer outcomes for the patient and could result in increased complaints [58]; furthermore, evidence suggests that locums are already more vulnerable to complaints than their permanent counterparts [3]. A meta‐summary of patient experiences when seeing multiple clinicians found that patients experience continuity of care as security and confidence rather than seamlessness [47]. We found that locums were sometimes perceived as overly cautious or lacking in confidence, which could weaken the notion of continuity of care and result in less satisfying outcomes for patients. Patients in our study described feeling safe when they saw a doctor who knew and understood their condition. This study provides further evidence that patients conceptualise safety in terms of how healthcare professionals make them feel, with feelings of safety being intrinsically linked to having trust and confidence in their care [31]. Patients' perceptions and observations with regard to the qualities and skills of locums (such as seeking advice from a senior GP or being unfamiliar with computer systems or offering investigations) shaped feelings of safety.

## 6 Conclusion

This study has shown that locum working can have implications for how patients access and experience care, how doctors deliver care and for patient−doctor relationships. Continuity of doctor−patient relationship was unlikely regardless of contractual status. However, patients with long‐term, complex or serious conditions value continuity of care and may avoid or delay seeking care if locums were their only option. Locums are not necessarily interchangeable with permanent members of the healthcare team who are familiar with their environments. Doctor familiarity with the patient, team and organisation can have implications for patients' outcomes, and whether or not the doctor is a locum should be communicated to patients to enable them to make informed choices about their care.

### 6.1 Strength and Limitations

To the author's knowledge, this is the largest qualitative study to explore locum working and quality and safety and the first study of its kind to explore patient perceptions and experiences of receiving care from locum doctors. However, it is possible that our sample may have been skewed towards patients who had negative perceptions and experiences of locum doctors, although patient perspectives were generally positive. We found that patients with long‐term and serious conditions were more likely to want continuity of care; however, we did not sample for patients with long‐term conditions meaning we may not have captured the views of patients who were most impacted by locum working. Also, some questions were related to participants' previous experiences, for which they needed to recall past situations. As a result, bias may have been introduced due to the unreliability of memory.

## Author Contributions

**Jane Ferguson:** conceptualisation, investigation, methodology, formal analysis, writing–original draft, writing–review and editing, visualisation. **Gemma Stringer:** conceptualisation, investigation, methodology, formal analysis, writing–review and editing, visualisation. **Kieran Walshe:** conceptualisation, investigation, methodology, formal analysis, writing–review and editing, visualisation. **Ailsa Donnelly:** conceptualisation, investigation, methodology, formal analysis, writing–review and editing, visualisation. **Christos Grigoroglou:** conceptualisation, writing–review and editing. **Thomas Allen:** conceptualization, formal analysis, writing–review and editing. **Evangelos Kontopantelis:** conceptualisation, writing–review and editing. **Darren M. Ashcroft:** conceptualisation, writing–review and editing.

## Conflicts of Interest

The authors declare no conflicts of interest.

## Supporting information

Supporting Information.

## Data Availability

The data are not publicly available due to privacy or ethical restrictions.
