# Supplementary material for: ‘None of Them Know Me’: A Qualitative Study of the Implications of Locum Doctor Working for Patient Experience
Source: Health Expect. 2024 Aug 1;27(4):e14156. doi: 10.1111/hex.14156 (PMC11292670; doi:10.1111/hex.14156)
Supplement: Supplementary file 1 — Supporting Information. [file HEX-27-e14156-s001.docx]

**Consolidated criteria for reporting qualitative studies (COREQ): 32-item checklist**

Tong A, Sainsbury P, Craig J. Consolidated criteria for reporting qualitative research (COREQ): a 32-item checklist for interviews and focus groups. International Journal for Quality in Health Care. 2007. Volume 19, Number 6: pp. 349 – 357

HEX-2024-6135 - ‘None of them know me’: a qualitative study of the implications of locum doctor working for patient experience

| **No. Item** | **Guide questions/description** | **Reported on Page #** |
| --- | --- | --- |
| **Domain 1: Research team and reﬂexivity** |  |  |
| *Personal Characteristics* |  |  |
| 1. Inter viewer/facilitator | Which author/s conducted the interview or focus group? | #4  Two members of the patient and public involvement forum conducted the patient focus groups supported by two authors. Three authors conducuctued one-to-one interviews with patients, locums and healthcare professionals who worked with locums. |
| 2. Credentials | What were the researcher’s credentials? | #1 Authors details and credentials. |
| 3. Occupation | What was their occupation at the time of the study? | #1 Authors details and credentials |
| 4. Gender | Was the researcher male or female? | Two members of our PPI forum were male and one was female.  Two authors who collected data were female and one was male. |
| 5. Experience and training | What experience or training did the researcher have? | The three authors who collected data have undertaken training in qualitative research methodologies and have extensive qualitative research experience as well as experience with this methodology. |
| *Relationship with participants* |  |  |
| 6. Relationship established | Was a relationship established prior to study commencement? | No. |
| 7. Participant knowledge of the interviewer | What did the participants know about the researcher? e.g. personal goals, reasons for doing the research | Participant information sheets were shared with participants detailing the purpose of the research. Participants knew which organisation the researchers worked for and who was funding the research. |
| 8. Interviewer characteristics | What characteristics were reported about the inter viewer/facilitator? e.g. Bias, assumptions, reasons and interests in the research topic. | Two of the authors had previously carried out research on the topic. The research team, and the PPI forum, worked reflexively discussing their personal biases and their potential impact on the research at regular meetings throughout the data collection and analysis period. |

| **Domain 2: study design** |  |  |
| --- | --- | --- |
| *Theoretical framework* |  |  |
| 9. Methodological orientation and Theory | What methodological orientation was stated to underpin the study? e.g. grounded theory, discourse analysis, ethnography, phenomenology, content analysis. | #4 Reflexive thematic analysis and abductive analysis. |
| *Participant selection* |  |  |
| 10. Sampling | How were participants selected? e.g. purposive, convenience, consecutive, snowball | #4 Purposive sampling. |
| 11. Method of approach | How were participants approached? e.g. face-to-face, telephone, mail, email | Email |
| 12. Sample size | How many participants were in the study? | #4 130 in total |
| 13. Non-participation | How many people refused to participate or dropped out? Reasons? | n/a |
| *Setting* |  |  |
| 14. Setting of data collection | Where was the data collected? e.g. home, clinic, workplace | Data were collected virtually in a place convenient to participants. Participants did not disclose their whereabouts. |
| 15. Presence of non-participants | Was anyone else present besides the participants and researchers? | No. |
| 16. Description of sample | What are the important characteristics of the sample? e.g. demographic data, date | #3 See Table 1 |
| *Data collection* |  |  |
| 17. Interview guide | Were questions, prompts, guides provided by the authors? Was it pilot tested? | #4 Three semi-structured interview and focus group guides were developed for use with patients, locums and health professionals working with locums. |
| 18. Repeat interviews | Were repeat inter views carried out? If yes, how many? | n/a |
| 19. Audio/visual recording | Did the research use audio or visual recording to collect the data? | #4 Audio recordings were made. |
| 20. Field notes | Were ﬁeld notes made during and/or after the interview or focus group? | Field notes were made during and after focus groups. |
| 21. Duration | What was the duration of the inter views or focus group? | #4 Focus groups typically lasted an hour while interviews varied in length from 23 to 171 minutes. |
| 22. Data saturation | Was data saturation discussed? | n/a The notion of data saturation is not consistent with the values and assumptions of reflexive thematic analysis. |
| 23. Transcripts returned | Were transcripts returned to participants for comment and/or correction? | No. |
| **Domain 3: analysis and ﬁndings** |  |  |
| *Data analysis* |  |  |
| 24. Number of data coders | How many data coders coded the data? | #4 Three authors coded the data. |
| 25. Description of the coding tree | Did authors provide a description of the coding tree? | #4 Coding summary is provided. |
| 26. Derivation of themes | Were themes identiﬁed in advance or derived from the data? | Derived from the data. |
| 27. Software | What software, if applicable, was used to manage the data? | #4 Nvivo |
| 28. Participant checking | Did participants provide feedback on the ﬁndings? | No |
| *Reporting* |  |  |
| 29. Quotations presented | Were participant quotations presented to illustrate the themes/ﬁndings? Was each quotation identiﬁed? e.g. participant number | #5-11  Quotations were presented and identified with a participant number. We also included whether they were a patient, a locum or another type of healthcare professional. |
| 30. Data and ﬁndings consistent | Was there consistency between the data presented and the ﬁndings? | #5-11 Yes, there was consistency between the data presented and the findings. |
| 31. Clarity of major themes | Were major themes clearly presented in the ﬁndings? | #5-11 Four major themes were presented. |
| 32. Clarity of minor themes | Is there a description of diverse cases or discussion of minor themes? | #5-11 While there was discussion of diverse cases, there was no descriptions of minor themes. |
